# Double-focusing mixing jet for XFEL study of chemical kinetics

**DOI:** 10.1107/S160057751401858X

**Published:** 2014-10-07

**Authors:** Dingjie Wang, Uwe Weierstall, Lois Pollack, John Spence

**Affiliations:** a Department of Physics, Arizona State University, PO Box 871504, Tempe, AZ 85287-1504, USA; b School of Applied and Engineering Physics, Cornell University, 254 Clark Hall, Ithaca, NY 14853, USA

**Keywords:** time-resolved diffraction, mixing jet, sample injection, chemical kinetics

## Abstract

Several liquid sample injection methods have been developed to satisfy the requirements for serial femtosecond X-ray nanocrystallography, which enables radiation-damage-free determination of molecular structure at room temperature. Time-resolved nanocrystallography would combine structure analysis with chemical kinetics by determining the structures of the transient states and chemical kinetic mechanisms simultaneously. A windowless liquid mixing jet device has been designed for this purpose. It achieves fast uniform mixing of substrates and enzymes in the jet within 250 µs, with an adjustable delay between mixing and probing by the X-ray free-electron laser beam of up to 1 s for each frame of a ‘movie’. The principle of the liquid mixing jet device is illustrated using numerical simulation, and experimental results are presented using a fluorescent dye.

## Abstract 2

A novel method for time-resolved study of chemical kinetics using a windowless mixing jet at an X-ray free-electron laser (XFEL) is described and demonstrated. The short mixing time gives a time resolution of about 250 µs; the design introduces controllable delays after the initiation of a chemical reaction, allowing the possibility for detection of transient structures by an XFEL beam pulse. Applications may include time-resolved enzyme–substrate imaging or protein folding.

## 1. Introduction   

Kinetic studies of conformational changes of macromolecules provide valuable information on the function and dynamics of biomolecules. A thorough study of reaction kinetics requires knowledge of the transient state of molecules during conformational changes. This necessitates the investigation of molecular structure at various time points, as in time-resolved crystallography, often based on pump–probe methods [see Aquila *et al.* (2012 ▶) for an X-ray free-electron laser (XFEL) example and Moffat (2013 ▶) for a review and references to earlier work at synchrotrons]. Here we consider chemical processes (such as a substrate–enzyme interaction, or protein folding or unfolding) where the rapid mixing of two solutions initiates a reaction. The mixing time then sets the time resolution of the structural measurements, which will use femtosecond pulses of an X-ray laser. These short pulses outrun radiation damage, allowing the study of protein molecules or nanocrystals at room temperature, alleviating concerns of damage due to freezing (Chapman *et al.*, 2011 ▶). The nature of some conformational changes, *e.g.* protein folding or unfolding, calls for new experimental methods which access a wide range of time scales. Several techniques have been adopted in the past, such as photochemical triggering, temperature/pressure jump and rapid fluid mixing [see Pollack & Doniach (2009 ▶) for a review].

XFELs have opened up new opportunities for crystallography (Liu *et al.*, 2013 ▶) due to the ability to outrun radiation damage in a ‘diffract-before-destroy’ read-out mode, and may also allow diffraction measurements with very high time-resolution at room temperature where multiple copies of a sample can be provided [see Spence *et al.* (2012 ▶) for a review]. XFELs can provide 10^12^ photons per 50 fs hard-X-ray pulse, currently at a pulse repetition rate of 120 Hz. The requirements for sample delivery in XFEL experiments, such as high replenishment rate in a hydrated environment in vacuum (Weierstall *et al.*, 2012 ▶), thus pose challenges for existing closed-cell liquid mixing methods (Schmidt, 2013 ▶). Turbulent mixing (Cherepanov & De Vries, 2004 ▶) can achieve extremely fast mixing times, but high sample consumption limits its utility for most biological samples. The extremely short and fixed mix-to-probe delay time also limits its application to measure full reaction time courses. Microfluidic devices (Pabit & Hagen, 2002 ▶) can be ruled out due to the extremely bright XFEL beam which vaporizes any material in its path.

Here we demonstrate a liquid mixing jet device, which mixes two liquids inside a nozzle and then injects them as a free jet into vacuum to achieve fast mixing and an adjustable delay time, while addressing the requirements of XFEL experiments. A coaxial liquid flow structure is utilized for mixing two liquids, then a gas focusing mechanism is used to form a continuous thin liquid jet (DePonte *et al.*, 2008 ▶; Weierstall *et al.*, 2012 ▶) while avoiding nozzle clogging problems. Mixer structure and fabrication is described, followed by numerical simulations and experimental results using a fluorescent dye to measure the performance of the mixing nozzle.

## 2. Fluid mechanics and device design   

**Figure 1:**
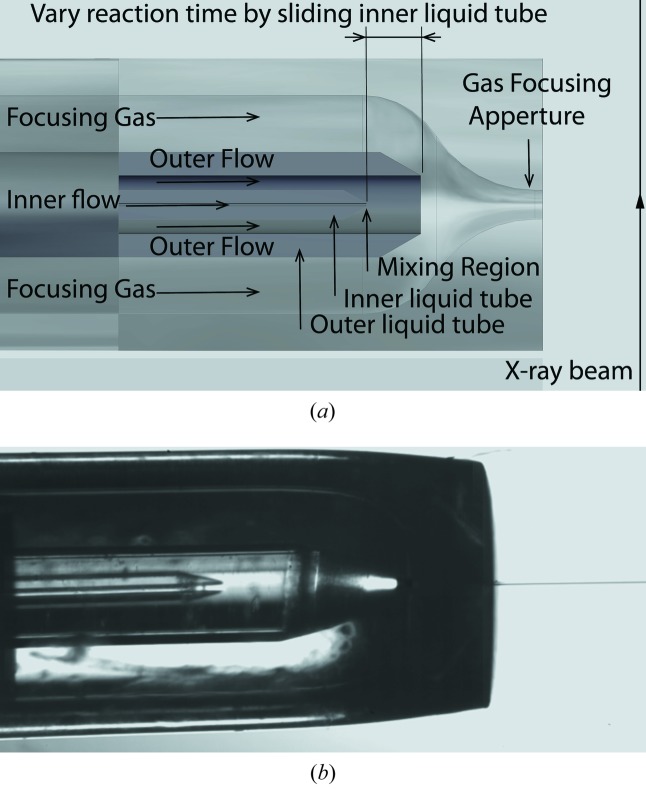
(*a*) Geometry and principle of the liquid mixing jet device. Two liquids, fed through the inner liquid line and the outer liquid line, mix at the end of the inner liquid line. After an adjustable delay, flowing between the end of the inner line and the end of the outer line, the flow goes through a gas focusing process and forms a thin jet. The delay time can be changed by changing the position of the inner liquid line relative to the outer liquid line. (*b*) Photograph of the actual device, showing the innermost capillary on-axis and the emerging jet.

A schematic of the mixing device is shown in Fig. 1(*a*) ▶, and the actual device in Fig. 1(*b*) ▶. The device consists of three coaxial telescopic tubes, namely an inner liquid tube with 20 µm inner diameter (ID) and 100 µm outer diameter (OD) containing liquid 1, an intermediate liquid tube with 200 µm ID and 360 µm OD containing liquid 2, and an outer gas focusing tube. By terminating the inner tube short of the outer two [which are about the same length, terminating in a gas dynamic virtual nozzle (GDVN) (DePonte *et al.*, 2008 ▶)], liquid 1 in the innermost tube emerges to meet and mix with liquid 2 before both liquids are gas focused and emerge from the GDVN nozzle as a free jet. Both liquid lines have a cone-shaped end for smooth fluid flow. The gas focusing aperture has an ID of 750 µm and an OD of 1000 µm, and its end is flame melted and formed to a specific shape for generating the gas focusing effect needed to form a free jet (Weierstall *et al.*, 2012 ▶).

As shown in Fig. 1 ▶, the solution of protein molecules or protein nano-crystals is fed through the inner liquid line, meeting with a solution of small molecules (reagents) which trigger the reaction when liquid 1 and 2 mix. The outer liquid 2 flows much faster than the inner liquid 1, causing a hydrodynamic focusing of the inner liquid flow at the exit of the inner capillary. The diameter of the inner flow decreases rapidly from 20 µm to approximately 1 µm, providing a short diffusion distance for reagents from the outer flow into the inner flow and therefore a short mixing time.

After the combined liquid flow leaves the end of the outer liquid line into vacuum, it passes through a gas focusing aperture and is accelerated by the focusing gas to form a free liquid jet with a diameter of 3–7 µm. The focusing is consistent with conservation of the product of area *A* and velocity *V* for incompressible flow. The device is therefore double-focusing. The free jet travels at a speed of about 10 m s^−1^ and remains continuous for several hundreds of micrometers, before breaking up by a necking instability into small droplets, similar to a Rayleigh jet (see DePonte *et al.*, 2008 ▶). The XFEL beam probes the jet in the continuous region, rather than the droplet region.

For different biomolecular processes, different mix-to-probe delay times are required to access the varied kinetic time scales of interest. For a specific process, measurements at different time points are needed to sample different transient states. An adjustable delay time is achieved by changing the distance between the end of the inner liquid line and the end of the outer liquid line. Since the liquid flow speed is extremely fast after the liquid leaves the end of the outer liquid line and forms a jet, and the mixing starts and finishes right after the end of the inner liquid line, it is reasonable to consider the mix-to-probe delay time as the time the liquid flow takes to travel from the end of the inner liquid line to the end of the outer liquid line. (Reverse diffusion of the much larger species in the inner line into the outer is negligible.) By changing the position of the inner liquid line relative to the outer liquid line, the time for the liquid to travel this distance can be varied to insert the desired time delay. For a typical flow rate for this device (*F* = 0.05 µl min^−1^ for inner flow, and *F* = 100 µl min^−1^ for outer flow), the velocity *V* = 2*F*/*A* = 30 mm s^−1^ at the center of Newtonian flow, and the delay time is adjustable in the range 10–1000 ms.

## 3. Experiments and simulations   

**Figure 2:**
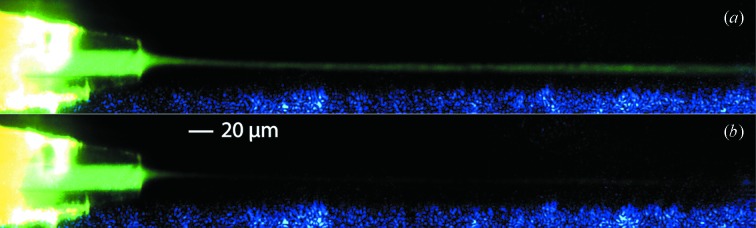
This fluorescence experiment illustrates the mixing process. The field of view shows only the innermost capillary tube at the ‘Mixing Region’ indicated in Fig. 1 ▶. (*a*) Fluorescent dye in the inner line, water in the outer line. (*b*) Fluorescent dye in the inner line, quencher in the outer line. Quenching represents the mixing process. A blue background is seen along the lower edge of each image.

Fluorescence experiments were carried out to demonstrate the fluid dynamics of the mixing process as shown in Fig. 2 ▶. The fluorescent dye sulforhodamine 101 solution was fed through the inner liquid line with a syringe pump at a flow rate 0.05 µl min^−1^, stimulated by a 528 nm laser, while water is fed through the outer liquid line at a flow rate of 100 µl min^−1^ (Fig. 2*a* ▶). At this condition the inner flow is focused down to about 3 µm in diameter within 20 µm of axial travel. This focusing distance can be decreased further to 1 µm by using a higher flow rate for the outer flow and a lower flow rate for the inner flow. In Fig. 2(*b*) ▶, instead of water, a solution of sodium iodide was fed through the outer liquid line to quench the fluorescence upon mixing. This fast interaction occurs much more rapidly than diffusion, and so can be used to measure the time taken for the two fluids to diffuse and mix (reaction times between biomolecules are much longer). The picture shows that the fluorescence is completely quenched within 20 µm of axial travel. Based on the flow speed, the elapsed time is less than 1 ms. Hence the upper limit of the mixing time is 1 ms for this flow rate. Under these flow conditions, simulations (below) reveal that the actual mixing time is much shorter than this upper limit. An even shorter upper limit of the mixing time down to less than 300 µs can also be achieved by decreasing the flow rate of the inner fluid, and increasing the flow rate of the outer fluid.

Excessive dilution can lead to a low hit rate by the X-ray beam, depending on the gas focusing process. This hit rate can only be determined by trials at an XFEL. The mixed flow may support two different modes after gas focusing (Gañán-Calvo *et al.*, 2007 ▶) based on the geometry of the gas aperture and the gas pressure, among other parameters. The inner flow could mix completely with the outer flow and form a uniformly mixed liquid jet after gas focusing, or the inner material could remain in the center of the liquid jet and form an extremely thin column. In the first case the hit rate will decrease. In the second case there would not be a dramatic increase in the background since the liquid jet diameter remains a few micrometers.

**Figure 3:**
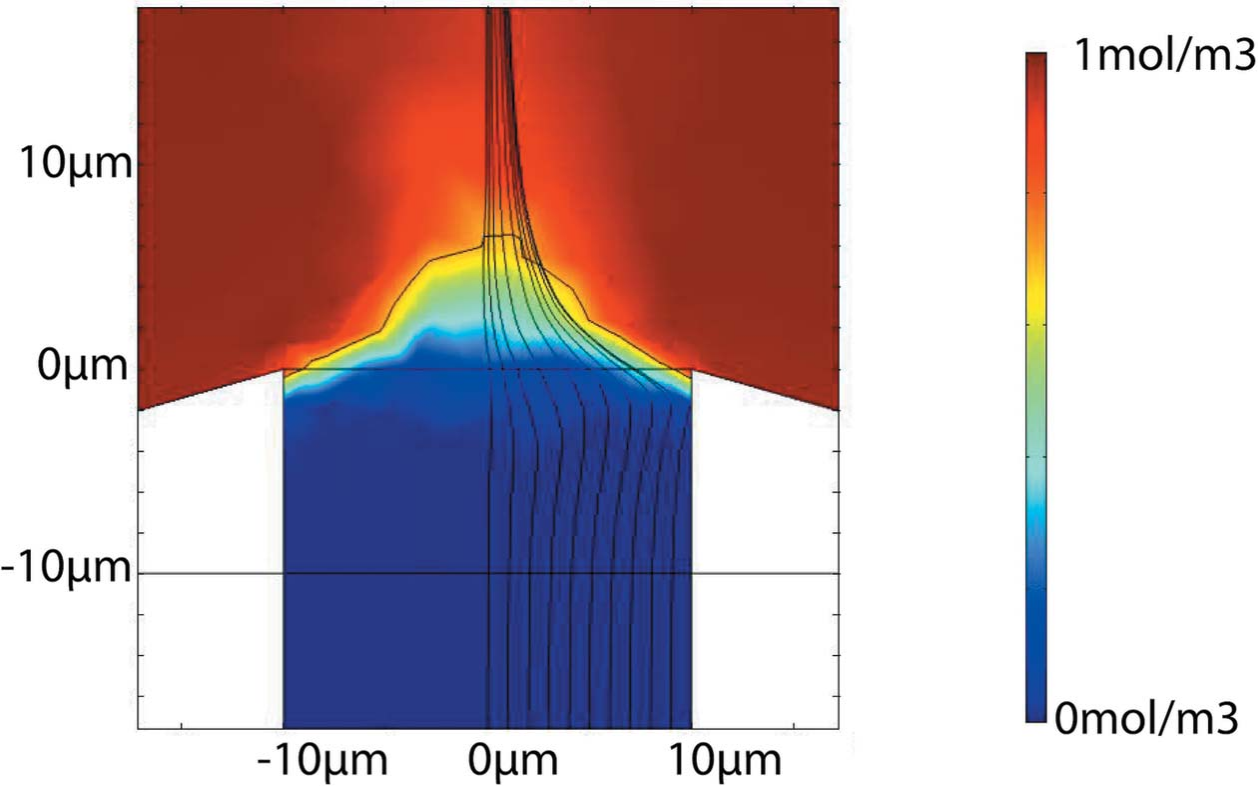
Numerical simulations illustrate the diffusion into the inner flow during the mixing process. The color scale shows concentration from 0 to 1 mol m^−3^. Contours represent concentrations of 0.7 mol m^−3^, the mixing criteria. Streamlines show the inner flow.

Numerical simulation of the mixing was carried out as shown in Fig. 3 ▶. A flow model was set up with the geometry of this device as two coaxial cylindrical tubes, with liquid solutions fed through both channels. The fluid dynamic process was simulated using the finite-element analysis method, with a particle transport mechanism embedded, to simulate the diffusion of molecules across the streamlines. The inner liquid line was fed with water at 0.1 µl min^−1^, the outer liquid line fed with water solution with 1 mol m^−3^ of solvent (with diffusion coefficient 3.0 × 10^−6^ cm^2^ s^−1^). If we define complete mixing when the inner flow reaches 70% of the concentration of the solute (0.7 mol m^−3^), then a contour of the mixing criteria can be drawn, as shown in Fig. 3 ▶. The mixing time can be defined as the standard deviation of the time every streamline takes from upstream to crossing the mixing criteria contour (Park *et al.*, 2006 ▶). Calculations show the time to be about 150 µs.

## 4. Conclusion   

By combining the advantages of a microfluidic mixer and a gas dynamic virtual nozzle, the double-focusing design of a liquid mixing jet device presented here achieves fast and uniform mixing of two solutions on a molecular scale with time resolution of about 250 µm while keeping the sample consumption low. This design also satisfies the high replenishment rate and sample environment requirements for XFEL experiments, enables radiation-damage-free studies of chemical kinetics at room temperature, and can be directly adopted by the LCLS beamline for experiments on time-resolved studies of biomolecular interactions. Further development of the design will focus on improved time resolution and a decreased dilution ratio, by possibly using multiple inner liquid tubes. This may be needed to increase the hit rate under some operating conditions.

## References

[bb1] Aquila, A. *et al.* (2012). *Opt. Express*, **20**, 2706–2716. 10.1364/OE.20.002706 PMC3413412 22330507

[bb2] Chapman, H. N. *et al.* (2011). *Nature (London)*, **470**, 73–77.

[bb3] Cherepanov, A. V. & De Vries, S. (2004). *Biochim. Biophys. Acta*, **1656**, 1–31. 10.1016/j.bbabio.2004.02.006 15136155

[bb4] DePonte, D. P., Weierstall, U., Schmidt, K., Warner, J., Starodub, D., Spence, J. C. H. & Doak, R. B. (2008). *J. Phys. D*, **41**, 195505.

[bb5] Gañán-Calvo, A. M., González-Prieto, R., Riesco-Chueca, P., Herrada, M. A. & Flores-Mosquera, M. (2007). *Nat. Phys.* **3**, 737–742.

[bb6] Liu, W. *et al.* (2013). *Science*, **342**, 1521–1524.

[bb7] Moffat, K. (2013). *Philos. Trans. R. Soc. B*, **369**, 20130568–20130572. 10.1098/rstb.2013.0568 PMC4052877 24914168

[bb8] Pabit, S. A. & Hagen, S. J. (2002). *Biophys. J.* **83**, 2872–2878. 10.1016/S0006-3495(02)75296-X PMC1302371 12414719

[bb9] Park, H. Y., Qiu, X., Rhoades, E., Korlach, J., Kwok, L. W., Zipfel, W. R., Webb, W. W. & Pollack, L. (2006). *Anal. Chem.* **78**, 4465–4473. 10.1021/ac060572n 16808455

[bb10] Pollack, L. & Doniach, S. (2009). *Methods Enzymol.* **469**, 253–268. 10.1016/S0076-6879(09)69012-1 20946793

[bb11] Schmidt, M. (2013). *Adv. Condens. Matter Phys.* **2013**, 1–10.

[bb12] Spence, J. C., Weierstall, U. & Chapman, H. N. (2012). *Rep. Prog. Phys.* **75**, 102601. 10.1088/0034-4885/75/10/102601 22975810

[bb13] Weierstall, U., Spence, J. C. & Doak, R. B. (2012). *Rev. Sci. Instrum.* **83**, 035108. 10.1063/1.3693040 22462961

